# A Metabolomics Approach Uncovers Differences between Traditional and Commercial Dairy Products in Buryatia (Russian Federation)

**DOI:** 10.3390/molecules23040735

**Published:** 2018-03-22

**Authors:** Lin Pan, Jie Yu, Zhihui Mi, Lanxin Mo, Hao Jin, Caiqing Yao, Dongyan Ren, Bilige Menghe

**Affiliations:** 1 Key Laboratory of Dairy Biotechnology and Engineering, Education Ministry of China, Inner Mongolia Agricultural University, Huhhot 010018, China; panlin_1988@163.com (L.P.); yujie8301@sina.com (J.Y.); nmgzhmi@163.com (Z.M.); mlanxin1121@163.com (L.M.); jinhao94@126.com (H.J.); 18848110382@163.com (C.Y.); pl_thyme99@emails.imau.edn.cn (D.R.); 2 Key Laboratory of Dairy Products Processing, Ministry of Agricultural, Department of Food Science and Engineering, Inner Mongolia Agricultural University, Hohhot 010018, China

**Keywords:** dairy product, metabolomics, ultra-performance liquid chromatography-quadrupole-time of flight mass spectrometry, statistical analysis

## Abstract

Commercially available and traditional dairy products differ in terms of their manufacturing processes. In this study, commercially available and traditionally fermented cheese, yogurt, and milk beverages were analyzed and compared. The metabolomic technique of ultra-performance liquid chromatography-quadrupole-time of flight mass spectrometry (UPLC-Q-TOF) in the MS^E^ mode was used in combination with statistical methods, including univariate analysis and chemometric analysis, to determine the differences in metabolite profiles between commercially and traditionally fermented dairy products. The experimental results were analyzed statistically and showed that traditional and commercial dairy products were well differentiated in both positive and negative ion modes, with significant differences observed between the samples. After screening for metabolite differences, we detected differences between traditional milk beverages and yogurt and their commercial counterparts in terms of the levels of compounds such as l-lysine, l-methionine, l-citrulline, l-proline, l-serine, l-valine and l-homocysteine, and of short peptides such as Asp-Arg, Gly-Arg, His-Pro, Pro-Asn. The greatest difference between commercially available and traditional cheese was in the short peptide composition, as commercially available and traditional cheese is rich in short peptides.

## 1. Introduction

The dairy industry has developed rapidly in recent years. Dairy products are a fundamental component of most people’s daily diets, and dairy products account for an increasing proportion of people’s caloric intake. Today, fermented food accounts for nearly 25–30% of the typical diet worldwide [1]. The dairy industry has also been a bright spot in the food industry due to its huge potential market and high rate of growth. Among dairy products, traditional fermented dairy products, such as fermented milk curd, cheese and yogurt are very rich in nutrients. Russia is a country with a long history and Buryatia is one of the member states of the Russian Federation. Due to their lifestyle the Mongolian people in Buryatia have abundant experience in making traditional dairy products. Jie et al. collected traditional dairy products including cheese from Buryatia and other parts of Russia. A total of 599 strains of lactic acid bacteria belonging to seven genera and 30 species were isolated. The dominant strains are Lactobacillus helveticus and Lactobacillus plantarum. The results demonstrate that the traditional dairy products from Russia have a complex lactic acid bacteria composition [2].

In traditional dairy products, *Lactobacillus* and yeast play important roles in determining flavor and aroma. The morphological diversity and variety of these microorganisms indicate a complex and stable biological environment [3,4,5]. However, due to a lack of in-depth research, certain deficiencies remain in terms of the flavor, texture and aroma of dairy products. Commercially available dairy products are produced according to the fermentation characteristics of commercial strains screened from traditional fermented dairy products having good characteristics. These optimized strains have been screened for stability and fermentation capability to obtain commercially viable dairy products. Dairy products fermented from a commercial starter culture are popular due to their unique flavor and high stability [6].

Fermented dairy products, which are consumed around the world, are produced by interactions among microorganisms. Lactic acid bacteria are commonly used in the starter culture. *Koji*, a fungus that has been consumed in Japan for over a thousand years, is used in the traditional Japanese fermented beverage *amazake* [7]. In one study, the components and functional compounds in *koji* were identified and analyzed using capillary electrophoresis time of flight mass spectrometry (CE-MS). The aim of this study was to explore the major differences between *koji* containing *Lactobacillus sakei* UONUMA and common *koji amazake* by means of metabolomics analysis. More than 300 compounds, including sugars, amino acids, organic acids and B-complex vitamins, were detected in *koji amazake*. Finally, 13 types of sugars, including two unknown trisaccharides, were identified. There was no significant difference in the bulk composition of the two *koji amazake* samples. However, lactic acid, vitamins B_2_ (riboflavin), B_3_ (nicotinic acid and nicotinamide), and B_6_ (pyridoxine) were significantly increased in *koji amazake* supplemented with lactic acid bacteria (LAF-*amazake*), while glutamine was decreased. LAF-*amazake* also produced the well-known neurotransmitter acetylcholine [7]. Metabolomics is widely used in modern food science and microbiology, in particular to address several systemic issues related to the safety, quality and nutritional status of food in studies of food quality, nutrition, and food safety [8]. Researchers have also applied metabolomics to fermented foods to observe the metabolomic changes during fermentation, which aids prediction of the organoleptic and nutritional qualities of the final product [9].

Metabolomic methods offer significant advantages for the identification of metabolites. Metabolomics can be used as an effective means of identifying biomarkers and applied to the protection of dairy products with regional characteristics unique to specific areas.

This technology mainly includes three part of sample pretreatment, metabolite determination and data analysis. The determination of metabolites is the core of metabolomics analysis. The three commonly used analytical methods of metabolomics are NMR, LC-MS and GC-MS. These analytical methods can both identify the structure of the metabolite and determine the concentration of the molecule [10,11]. After the data was collected, the original spectra from different analysis platforms were preprocessed. Combined with multivariate statistics and bioinformatics methods, the data were analyzed to find out the metabolic differences and biomarkers.

The correlation between the composition and metabolic status of milk can be determined through a combination of high-resolution nuclear magnetic resonance (NMR) spectroscopy and gas chromatography-mass spectrometry, and allows systematic study of the diverse range of physiological metabolites present in dairy cows during lactation [12]. Using metabolomic techniques, 44 different milk metabolic differences were identified and specific biomarkers, such as acetone and β-hydroxybutyrate, were significantly associated with metabolic stress in dairy cows during early lactation. Further metabolomic analysis may support selection of cows that can tolerate early lactation metabolic stress [13]. During the fermentation of soybeans into *meju*, 22 metabolites were identified as biomarkers of metabolic pathways [14].

The most commonly used metabolomic techniques are high-resolution proton NMR spectroscopy (^1^H-NMR), liquid chromatography-mass spectrometry (LC-MS), gas chromatography-mass spectrometry (GC-MS), and capillary electrophoresis-mass spectrometry (CE-MS) [9,15].

These techniques have been successfully utilized for determination of metabolites in fermented dairy products during fermentation, maturation and storage [1,9,16,17]. The ^1^H-NMR technique was used to identify changes in non-volatile metabolites at six time points during the fermentation of soybean paste [18]. To study changes in the metabolites present during the fermentation of the soybean pastes *doenjang* and *cheonggukjang*, GC-MS was applied [19,20]. Multivariate analysis with gas chromatography/time-of-flight mass spectrometry (GC-MS) was performed on 13 cheeses, including six cheddar, six Gouda and one Parmigiano-Reggiano cheese, and the results showed that principal component analysis (PCA) based on metabolomics can identify the distinctive sensory characteristics associated with maturation, and GC-MS can also be used for modeling the sensory predictive of cheese [21]. Recent reports show that metabolomics analysis has made a significant contribution to research on milk and dairy products. Han et al. established a selection-specific assay involving ultra-high-performance liquid chromatography–tandem mass spectrometry (UPLC-MS/MS). Their method rapidly identified a total of 38 veterinary antibiotic residues, across six different classes, in raw milk. When this method was applied to 25 samples of raw milk collected on site, traces of four veterinary antibiotic residues were detected in three samples [22].

In this study, cheese, yoghurt and milk beverages, including traditional and commercially available dairy products, were subjected to analysis. The ultra-performance liquid chromatography-quadrupole-time of flight mass spectrometry (UPLC-Q-TOF MS) in the MS^E^ mode was used to analyze traditional and commercial dairy products. This technique combines univariate and chemometric data processing methods to analyze differences in the metabolite profiles of dairy products from different sources. The results of this study provide reference data for understanding the compositional differences between traditional and commercial dairy products.

## 2. Results and Discussion

### 2.1. Metabolomics Data Analysis

After three series of samples were processed, base peak ion (BPI) chromatograms were obtained in positive and negative electrospray ionization (ESI^+^/ESI^−^) scanning modes under optimized LC-MS conditions.

Under the optimized LC-MS conditions, a small-molecule metabolite profile of each sample was established. First, the BPI chromatograms of all samples were visually inspected, and a preliminary analysis of the overlapping BPI images in the three groups of samples was conducted (Figures S1–S3). The chromatographic peaks were well separated, with almost all of them reaching the baseline separation; the retention time (RT) and distribution of the main peaks showed good reproducibility. No differences in the spectra of the groups were identified from the BPI images. To identify the metabolic characteristics of each population, their omics features were determined using a combination of unidimensional and multidimensional statistics.

Chemometrics is an important tool for analyzing metabolomic differences among samples, as it enables identification of metabolites associated with specific conditions. To investigate the differences in metabolites among the three groups of samples, two multivariate statistical analyses, unsupervised PCA and orthogonal partial least squares discriminant analysis (OPLS-DA), were performed on the dairy product data obtained under the ESI^+^ and ESI^−^ scanning modes using the MassLynx software (version 4.1, Waters Corporation, Milford, MA, USA).

**Figure 1:**
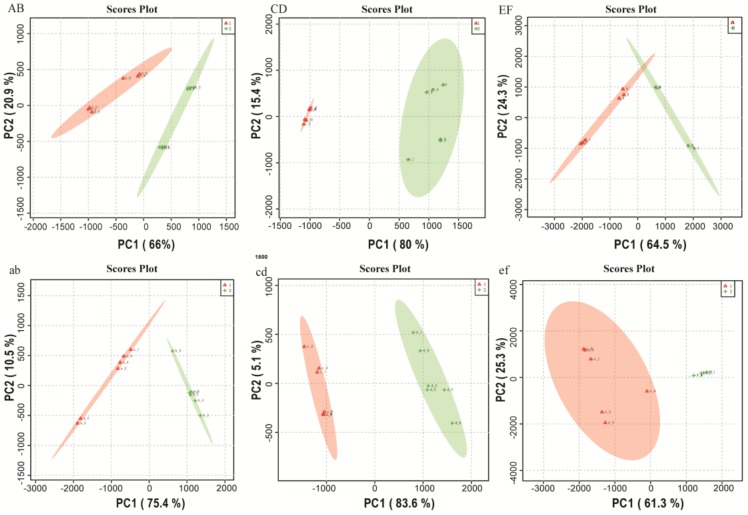
Principal component analysis (PCA) score plots (S plots) of three groups of samples. Data were obtained in positive electrospray ionization (ESI+: **AB**, **CD**, **EF**) and negative electrospray ionization (ESI^−^: **ab**, **cd**, **ef**) ion modes.

Because the samples were independent, the PCA score plots (Figure 1) of the dairy product data obtained under the ESI^+^/ESI^−^ scanning mode could be aggregated into groups, which differed markedly and were clearly separable. The plots show the same results as actually divided into two groups between commercially available and traditional dairy products, and there were statistically significant differences between the commercial and traditional products within each of the three groups of dairy products sampled in terms of their metabolite profiles.

**Figure 2:**
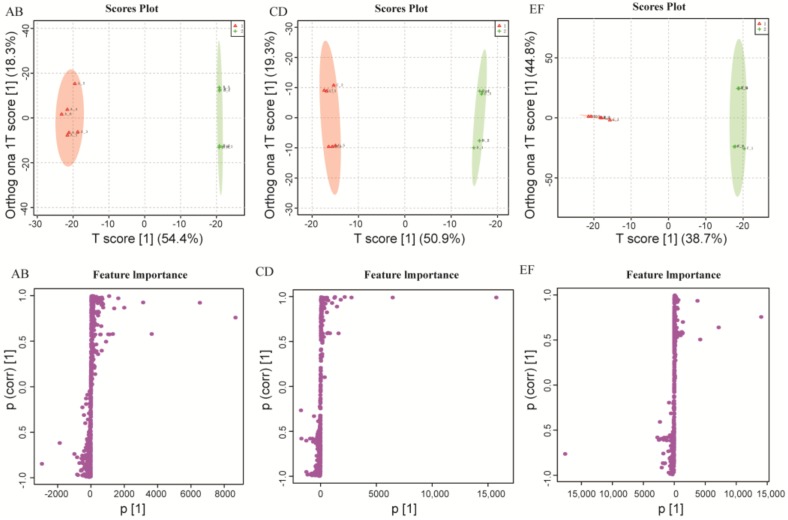
Orthogonal partial least squares discriminant analysis (OPLS-DA): S-plots of the data for three groups of samples (**AB**, **CD**, **EF**) obtained in ESI^+^ ion mode.

OPLS uses orthogonal signal correction (OSC) to filter out information that has no relationship with the quantity matrix (Y) in the independent variable matrix (X), that is, random noise. Thus, OPLS-DA can better identify differences between groups of samples and thereby improve a model’s effectiveness and analytical power. Mass spectrometry data of the three groups of dairy product samples were analyzed using OPLS-DA to obtain S-plots of each ESI^+^ and ESI^−^ pair (Figure 2 and Figure S4).

The OPLS-DA data (Figure 2) show that traditional dairy products are well distinguished from commercial dairy products in both ESI^+^ and ESI^−^ modes. The fitness power and predictive power are represented by R^2^Y and Q^2^, respectively. In ESI^+^ mode, R^2^Y = 0.994 and Q^2^ = 0.978 for group AB; R^2^Y = 0.992 and Q^2^ = 0.975 for group CD; and R^2^Y = 0.763 and Q^2^ = 0.684 for group F. In ESI^−^ mode, R^2^Y = 0.965 and Q^2^ = 0.88 for group AB; R^2^Y = 0.997 and Q^2^ = 0.892 for group CD; and R^2^Y = 0.934 and Q^2^ = 0.819 for group EF. The R^2^Y and Q^2^ value of the six models are relatively high, showing that they are well differentiated and have good predictive power, and that the samples comprising each have significant metabolic differences based on their OPLS-DA scores.

On the S-plots, each point represents a metabolite. The metabolite has greater divergence between the two groups farther from the center axis. It is apparent from the S-plots that the metabolite profiles of the commercially and traditionally fermented dairy products are distinctly different. By reference to the p values of the OPLS-DA data, and by using the variable importance in projection (VIP) method and assessing other parameters, differences in metabolites between commercially and traditionally fermented dairy products can be better explained.

### 2.2. Comparison of Metabolite Profiles Between the Different Groups

MassLynx software (version 4.1) was used, in combination with a database (Kyoto Encyclopedia of Genes and Genomes; KEGG; www.genome.jp/kegg), ChemSpider (www.chemspider.com), Human Metabolome Database (HMDB; www.hmdb.ca) and METLIN (www.metlin.scripps.edu), to identify metabolites within the MS^E^ data, which contain precursor and corresponding fragment ion information.

The ESI^+^ and ESI^−^ scanning mode data for group AB contained a total of 23,595 compounds, among which 22,123 metabolites were in positive ion scanning and 1472 metabolites were in negative ion scanning state. A total of 23,595 compounds were detected in group CD, among which there were 743 positive ions and 20,578 negative ions. A total of 21,404 metabolites were detected in group EF, including 1752 positive ions and 1652 negative ions.

According to multivariate statistical analysis combined with three criteria. The result is shown in Table 1, which indicates that the compounds identified in the two types of fermented milk samples were mainly amino acids, short peptides, lipids, nucleic acids, carbohydrates, and compounds related to energy metabolism.

A total of 58 compounds were identified, as listed in Table 1, of which 22 were amino acids such as l-lysine, l-methionine, l-citrulline, l-proline, l-lysine, l-serine, l-valine, and l-homocysteine. Sixteen peptides were identified, including Asp-Arg, Gly-Arg, His-Pro, and Pro-Asn. Other classes of metabolites that were well-represented included lipids, nucleic acids, and compounds related to energy metabolism, such as hexadecenoic acid, purine, and trimethylamine. A few of the compounds identified are involved in the biosynthesis of secondary metabolites, metabolism of cofactors and vitamins, membrane transport and gene translation. The proportion of amino acids, such as l-lysine, l-citrulline, l-proline, d-fructose, and purine, was significantly higher in the metabolites of traditional versus commercially available dairy products.

In group CD, the most abundant compounds were amino acids and short peptides. Pretyrosine, aldosterone, β-alanyl-l-arginine, *O*-acetyl-l-serine, *N*-formyl-l-methionine, phenylacetylglycine, l-phenylalanine, cystathionine, pyrimidine, threonine, l-cysteine, l-leucine, Cys-Ser, Gly-Phe, carnosine, Ser-Met, Pro-His, Met-Asn, Gln-His, Lys-Phe, and Pro-Asn were found in higher amounts in traditional versus commercial dairy products.

In group EF, the metabolites was less than that in group AB and CD, but in positive ion scan mode, it was observed that the differences metabolites of cheese was rich in short peptides. Aspartic acid, *N*-acetyl-l-cysteine, Asp-Val, Leu-Asp, Asp-Asp, Gln-Phe, Trp-Arg, methanesulfonic acid, benzoquinone, cytosine, l-proline, l-valine, 4-oxoproline 3-sulfino-l-alanine, phosphocreatine, and Ser-Trp were present in higher amounts in traditional dairy products than in commercially available dairy products, while Cys-Asn, His-Val, Lys-Lys, l-homoserine, alanyl-d-alanine, Thr-Val, l-cysteine, and Lys-Phe contents were higher than traditional dairy products.

## 3. Discussion

Different types of dairy products have different starters, which is also one of the important influencing factors of metabolites. Emerging technological and analytical metabolomics approaches have been widely used to study drug toxicity and its underlying mechanisms in microbial and plant research, animal models, food nutrition studies, and gene function investigations, as well as for disease diagnosis [12,23]. Metabolomics can be used to accurately identify amino acids, carbohydrates, organic acids, vitamins, peptides, nucleotides and other metabolites in complex samples.

### 3.1. Effects of Starter Diversity on the Metabolites of Dairy Products

In one study, *Lactobacillus rhamnosus* GG (Y-LGG) and *Bifidobacterium animalis* subsp. *lactis* BB12 (Y-BB12) were combined in yoghurt fermentation. The metabolites were measured by headspace solid phase microextraction-gas chromatography/MS (SPME-GC/MS) and ^1^H-NMR techniques. The final PCA and loading plot result shows that the metabolites of Y-LGG and Y-BB12 are clearly separated [24]. In addition, starters under nutrition stress can also lead to changes in metabolites. *Lactobacillus plantarum* WCFS1 was pre-cultured under elevated NaCl and low pH stress conditions and metabolomics analyses revealed that the presence of stress-adapted probiotics induced significant changes in the overall metabolite profile of yoghurt [25].

### 3.2. Amino Acids

In fermented dairy products, the type and quantity of amino acids are often used as important indicators of nutritional quality. Kang et al. increased the fermentation time of *meju* and identified metabolites responsible for the unique flavor and nutritional attributes of soybean foods, including amino acids, small peptides, nucleotides, urea cycle intermediates and organic acids based on the ultra-performance liquid chromatography-quadrupole-time of flight mass spectrometry (UPLC-Q-TOF MS) assay method. In that experiment, 22 metabolic compounds were identified as biomarkers of the metabolic pathways of *meju* fermentation [14]. In metabolomics research on *doenjang* fermentation, the concentrations of amino acids including alanine, threonine, glycine and serine increased between 140 and 160 days of fermentation, while those of leucine and isoleucine increased continuously from day 100 [19]. In PCA, 50% methanol extracts of a fermented soybean paste showed clear differences by fermentation time: the major compounds identified were isoleucine, leucine and tyrosine [18]. Amino acids identified in the present experiment included l-proline, l-methionine, l-citrulline, l-lysine, serine, valine, glycine, proline, and threonine, which are commonly found in fermented milk [16,17,21,26,27,28].

### 3.3. Peptides

Milk can be degraded into various metabolites by lactic acid bacteria, including aromatic compounds and peptides. Short peptides, including Asp-Arg, Gly-Arg, Pro-Asn and Arg-Cys were detected in the samples in our experiment. These metabolites have been shown to affect the texture and flavor of fermented dairy products.

In a previous study, 223 metabolites were identified in 10 different commercially available types of milk using a combination of liquid chromatography and gas chromatography. These metabolites included amino acids, lipids, carbohydrates, nucleotides, energy metabolites, vitamins, adjuvant factors, and short peptides [16]. During the fermentation process, casein is degraded into short peptides through the actions of protease and peptidase. Researchers have used metabonomics to determine that these short peptides may have beneficial effects on human health, such as lowering cholesterol, easing stress and exerting antioxidant activity [29]. Milk fermented by *Streptococcus thermophilus* and *Lactobacillus plantarum* can improve the functional and probiotic properties of yoghurt, as the milk protein degrades into a large number of short peptides and amino acids that have biological activities [27]. The most significant changes revealed by an NMR-PCA method during the 90-day fermentation of traditional Italian raw ewe’s milk cheese, Fiore Sardo, were that the carbohydrate, citric acid and lactic acid contents decreased at the beginning of maturation, while amino acids and smaller peptides formed toward the end of maturation [17].

### 3.4. Other Metabolites

Fermented milk, a popular and healthy food, is favored by a growing number of individuals, and the unique flavor that was formerly perceived as problematic is now widely accepted by consumers. Among metabolites, organic acids are an important determinant of yogurt flavor. Therefore, determination of the organic acid composition and content plays an important role in evaluating the nutritional value, aroma, flavor and biological activity of fermented milk products. Several organic acids in fermented products, including lactic acid, acetic acid, and citric acid have been reported as differential metabolites compared with non-fermented products through high-resolution magic angle spinning nuclear magnetic resonance (^1^H-RMAS-NMR) and GC-MS analyses [19,20,30]. Pisano et al. characterized the metabolites in Italian buffalo cheese and compared them with those of cow mozzarella. Lactic acid, citric acid, succinic acid, fumaric acid, glyceric acid, and gluconic acid have also been identified as differential metabolites using GC-MS [28].

Vitamins, amines and fatty acids are also common metabolites in fermented dairy products [21]. Saccharides such as galactose, maltose, ribose and myo-inositol have been identified in fermented dairy products, along with lactic acid dimers. Long-chain fatty acids, including stearic acid, oleic acid and palmitic acid, have also been observed [28].

## 4. Materials and Methods

### 4.1. Sample Collection

Commercially available samples study in this experiment was imported from Buryatia (Russian Federation) and purchased from a Chinese supermarket. Each of the commercially available dairy products had the same expiry date and were taken from the same store shelf. In order to avoid the influence of the season, all dairy products samples were purchased on the same day. In the selection of commercially available milk beverages, we choose samples containing only Lactobacillus casei as a starter. In the process of choosing yoghurt, we only selected the yogurt with Lactobacillus bulgaricus subsp. bulgaricus and Streptococcus thermophilus. When we selected the samples of cheese, only Lactococcus lactis as starters can be involved. Traditional dairy products were obtained from the residence of a Mongolian herdsman in Village of Tunka (Buryatia). Both commercially available dairy products and traditional dairy products have the same fermentation end-point, that is, the value of pH is equal to 4.5. Milk samples were collected into 50-mL sterile screw-top tubes, sealed and labeled, and preserved in a freezer at −80 °C. Commercial samples with a production date close to the date of collection were chosen, placed into sterile centrifuge tubes and stored at −80 °C for later use. The samples were divided into three groups: AB, comprising one commercial and one traditional homemade fermented milk beverage; CD, comprising one commercial yogurt and one traditional homemade fermented yogurt; and group EF, comprising one commercial and one traditional homemade fermented cheese.

### 4.2. Sample Preparation for UPLC-Q-TOF MS^E^ Analysis

Samples were thawed and centrifuged at 4500× *g* for 10 min, and then the fat layer was discarded. From the supernatant, 2 mL was collected, to which 14 mL acetonitrile was added, and the mixture was vortexed for 2 min. Ultrasonic extraction of the sample was conducted for 5 min, followed by 12,000× *g* high-speed centrifugation for about 15 min. Then, the supernatant was collected, concentrated through drying in a vacuum rotary enrichment apparatus, and reconstituted in 500 μL of 40% (*v*/*v*) acetonitrile. After reconstitution, the sample solution was filtered through a 0.22-μm water-insoluble microporous membrane into a vial, and preserved at −20 °C until testing.

### 4.3. UPLC-Q-TOF MS^E^ Analysis

An ACQUITY UPLC^®^/Xevo^®^ G2 Q TOF-MS^E^ (Waters Corporation, Milford, MA, USA) was used to extract MS spectral peaks of dairy products. Pre-treated milk samples were chromatographed using a C18-UPLC system. The column was HSS T3 (2.1 mm × 100 mm, 1.8 μm, Waters). Each sample infusion flow rate of 0.3 μL/min and the column was maintained at 35 °C. The injection volume is 4 μL.

As for ESI^+^ ion mode, the mobile phase A is an aqueous phase containing 0.1% formic acid (Sigma-Aldrich, St. Louis, MO, USA). Mobile phase B is an organic phase containing 0.1% formic acid in acetonitrile (Optima^®^ LC/MS-grade, Fisher, Grand Island, NY, USA). The elution gradient was as follows: initial, 90% A, 10% B (0–2 min); 90% A, 10% B (2–12 min); 60% A, 40% B (12–20 min); 30% A, 70% B (20–23 min); 30% A, 70% B (23–24 min).

In the scanning of ESI-ion mode, the mobile phase consisted of water (Millipore, Bedford, MA, USA) containing 10% acetonitrile (solution A) and 50% acetonitrile (solution B). The elution gradient was as follows: initial, 90% A, 10% B (0–2 min); 90% A, 10% B (2–12 min); 60% A, 40% B (12–20 min); 30% A, 70% B (20–23 min); 90% A, 10% B (23–24 min).

When the dairy products in this experiment were analyzed by metabolomics, the Q-TOF mass spectrometer carried out data scanning using the positive positive [electrospray ionization, ESI^+^] and negative [ESI^−^] ion modes. Source temperature, 100 °C; desolvation Temperature 150 °C; desolvation gas flow, 50 L/H(+) and 800 L/H(−), respectively; cone gas flow, 50 L/H, capillary 3 kV; cone voltage, 40 V; the acquisition scan range was from 50 to 2000 *m*/*z*; scan frequency 10.00 s.

In order to ensure the accuracy of data acquisition, leucineen-kephalin was used as the lock mass for positive ion mode (556.2771 [M + H]^+^) and negative ion mode (554.2615 [M − H]^−^).

### 4.4. UPLC-Q-TOF MS^E^ Data Processing and Multivariate Statistical Analyses

After LC-MS analysis of the fermented milk samples, the raw UPLC-Q-TOF MS^E^ data were imported into MassLynx software (ver. 4.1) which was employed for peak matching, peak alignment, peak extraction and normalization. After normalization and standardization of the original data, SIMCA-P + 11 (Umetrics AB, Umea, Sweden) was used for multivariate statistical analysis. The data were exported in .txt format from SIMCA-P + 11 and included the mass-to-charge ratio (*m*/*z*) and RT. Finally, six sets of data were obtained in positive ion detection mode. Negative ion data were obtained as described above. PCA and PLS-DA were used to identify differences in metabolite profiles among samples. Second, the PCA and PLS-DA data were analyzed using Metaboanalyst (www.metaboanalyst.ca). Finally, using multivariate statistical analysis combined with the three criteria, which are VIP value ≥ 2, fold-change ≥ 2 and *p*-value < 0.05, the most significant variance was selected. The structure of the screened differential metabolites were determined according to the *m*/*z*, RT, and fragment ion information.

### 4.5. Identification of Key Metabolites

Following the data analysis, the metabolites were screened according to their RT, *m*/*z* and fragment ion information using MassLynx softwere (ver. 4.1) online, which is linked to the commonly used metabolomic databases Kyoto Encyclopedia of Genes and Genomes (KEGG; www.genome.jp/kegg), ChemSpider (www.chemspider.com), Human Metabolome Database (HMDB; www.hmdb.ca) and METLIN (www.metlin.scripps.edu) [31]. The final data were reviewed according to relevant literature to determine the compounds present. Each aromatic compound was analyzed using the MetaboAnalyst (http://www.metaboanalyst.ca) and KEGG (http://www.genome.jp/kegg/pathway.html) databases to identify the metabolic pathways showing significant differences between the commercial and traditional products within each of the three groups of dairy products sampled.

## 5. Conclusions

In this study, we showed that metabolomics analysis combined with statistical methods has great potential for distinguishing between different types of dairy products with different starters from Buryatia in the Russian Federation. Metabolomic methods can be used to identify and analyze differenti compounds in fermented dairy products, which could serve as potential biomarkers. This study shows that metabolomics is an ideal technology for distinguishing among, and identifying compounds in, dairy products.

## Figures and Tables

**Table 1:** Metabolites showing a significant difference between samples in positive and negative ion modes.

RT (min)	*m/z*	Identities	MF	MP	Fold-Change	*p*-Value
Group AB/Positive						
0.8962	85.0224	4-Aminobutyraldehyde	C_4_H_9_NO	AA	0.42	2.38 × 10^−5^
0.7689	248.0213	4,4′-Sulfonyldianiline	C_12_H_12_N_2_O_2_S	AA	248,090	6.23 × 10^−5^
0.8486	163.0584	*N*-Acetyl-l-cysteine	C_5_H_9_NO_3_S	-	0.52	7.50 × 10^−5^
0.8739	145.0474	l-allysine	C_6_H_11_NO_3_	-	0.38	0.01876
0.9598	289.0916	Asp-Arg	C_10_H_19_N_5_O_5_	PP	0.0051876	3.63 × 10^−7^
0.9691	127.0359	*N*-Cyclohexylformamide	C_7_H_13_NO	-	0.07	0.00059
0.9945	254.1610	Hexadecenoic acid	C_16_H_30_O_2_	LL	0.02	1.31 × 10^−8^
1.5347	120.7750	Phenylacetaldehyde	C_8_H_8_O	AA	1.36	2.46 × 10^−5^
1.6726	100.0709	4-Methylpentanal	C_6_H_12_O	LL	12.84	1.76 × 10^−8^
2.6366	146.0578	l-Lysine	C_6_H_14_N_2_O_2_	AA/SM/LL/MT/CV/TL	3.15	4.14 × 10^−5^
2.6422	188.0694	homo-*cis*-Aconitate	C_7_H_8_O_6_	AA	3.97	2.96 × 10^−5^
3.7878	227.1748	Deoxycytidine	C_9_H_13_N_3_O_4_	NT	240,070	6.62 × 10^−7^
3.9296	246.1697	Methionyl proline	C_10_H_18_N_2_O_3_S	TL	191,130	0.00021
5.2484	141.0520	Histidinol	C_6_H_11_N_3_O	AA/SM	4.64 × 10^−7^	1.35 × 10^−6^
8.7712	230.1133	Ergothioneine	C_9_H_15_N_3_O_2_S	AA	9.16	1.83 × 10^−9^
5.2484	126.0279	Thymine	C_5_H_6_N_2_O_2_	NT	0.0001341	8.27 × 10^−9^
6.0582	120.0774	Purine	C_5_H_4_N_4_	NT/CH	286,000	2.64 × 10^−7^
8.2841	251.1497	Deoxyadenosine	C_10_H_13_N_5_O_3_	NT	197,920	4.11 × 10^−7^
9.4986	231.1697	Gly-arg	C_8_H_17_N_5_O_3_	PP	4.87 × 10^−6^	1.42 × 10^−6^
14.6231	274.2742	Lys-Lys	C_12_H_26_N_4_O_3_	PP	123.70	0.00381
15.2054	149.0212	l-Methionine	C_5_H_11_NO_2_S	AA/TL/SM	1.62 × 10^−6^	1.29 × 10^−5^
**Group AB/Negative**						
1.5263	252.0899	His-Pro	C_11_H_16_N_4_O_3_	PP	1.91 × 10^−7^	0.00020
1.6961	229.1568	Pro-Asn	C_9_H_15_N_3_O_4_	PP	4,255,800	2.97 × 10^−7^
2.3017	119.0502	Threonine	C_4_H_9_NO_3_	AA	2.59 × 10^−7^	2.52 × 10^−9^
2.6692	236.0936	Ala-Phe-Ala	C_12_H_16_N_2_O_3_	PP	3.33 × 10^−7^	3.00 × 10^−7^
1.0490	218.0679	*N*-Acetylserotonin	C_12_H_14_N_2_O_2_	AA	3.40 × 10^−7^	5.55 × 10^−6^
2.0917	109.0293	Aminophenol	C_6_H_7_NO	AA	3.78 × 10^−7^	5.44 × 10^−8^
4.6603	277.1568	Arg-Cys	C_9_H_19_N_5_O_3_S	PP	1,862,300	0.00020
1.5262	188.0940	Ethyl glutarate	C_9_H_16_O_4_	-	5.78 × 10^−7^	6.58 × 10^−6^
2.5673	140.1082	Ethyl furoate	C_7_H_8_O_3_	-	5.92 × 10^−7^	5.11 × 10^−6^
2.2441	158.1203	Pentyl butyrate	C_9_H_18_O_2_	-	1.40 × 10^−6^	3.29 × 10^−5^
0.7095	95.9522	Sodium propionate	C_3_H_5_NaO_2_	-	578,660	4.89 × 10^−5^
1.8233	144.0477	Octanoic acid	C_8_H_16_O_2_	-	504,840	0.00070
1.7620	175.0628	l-Citrulline	C_6_H_13_N_3_O_3_	AA/SM	371,400	0.00043
1.7515	115.0419	l-Proline	C_5_H_9_NO_2_	AA/SM/MT/TL	360,600	8.55 × 10^−5^
1.0344	305.0895	Thr-Trp	C_15_H_19_N_3_O_4_	PP	0.000104	6.20 × 10^−6^
1.5826	242.0134	Thymidine	C_10_H_14_N_2_O_5_	NT	0.000872	9.14 × 10^−9^
1.7400	180.0685	d-Fructose	C_6_H_12_O_6_	CH	604.44	1.32 × 10^−6^
1.5606	162.0562	Trimethylamine	C_3_H_9_N	EG/CH	0.002953	4.10 × 10^−7^
0.7924	197.0226	*N*-Hydroxy-l-tyrosine	C_9_H_11_NO_4_	SM	<0.003601	1.43 × 10^−5^
2.7630	204.0687	Ala-Asp	C_7_H_12_N_2_O_5_	PP	<0.004426	3.90 × 10^−5^
1.0189	190.0735	Asp-Gly	C_6_H_10_N_2_O_5_	PP	0.01	1.80 × 10^−7^
1.7732	252.0892	Ala-Tyr	C_12_H_16_N_2_O_4_	PP	140.78	0.00041
2.5732	263.1406	Asn-Met	C_9_H_17_N_3_O_4_S	PP	132.26	8.29 × 10^−7^
0.7738	149.0453	l-Methionine	C_5_H_11_NO_2_S	AA/TL/SM	0.011586	1.33 × 10^−5^
1.0465	146.0453	L-Lysine	C_6_H_14_N_2_O_2_	AA/SM/LL/MT/CV/TL	0.02	2.29 × 10^−5^
0.9995	129.0195	Isoquinoline	C_9_H_7_N	AA/SM	0.03	1.56 × 10^−9^
0.9995	220.0733	Met-Ala	C_8_H_16_N_2_O_3_S	PP	0.03	0.00019
1.4599	181.0513	Tyrosine	C_9_H_11_NO_3_	AA	0.04	2.68 × 10^−7^
1.4716	258.0065	His-Cys	C_9_H_14_N_4_O_3_S	PP	0.06	7.40 × 10^−7^
0.7334	105.0183	l-Serine	C_3_H_7_NO_3_	AA/SM	0.07	4.63 × 10^−5^
1.0926	117.0190	l-Valine	C_5_H_11_NO_2_	AA	0.07	1.43 × 10^−6^
1.4746	163.0403	*N*-Acetyl-l-cysteine	C_5_H_9_NO_3_S	-	0.09	1.96 × 10^−5^
1.4847	119.0499	Threonine	C_4_H_9_NO_3_	AA	0.10	2.38 × 10^−5^
1.4851	135.0452	l-Homocysteine	C_4_H_9_NO_2_S	AA/SM	0.11	6.19 × 10^−5^
1.3484	218.1040	Thr-Val	C_9_H_18_N_2_O_4_	PP	0.13	3.68 × 10^−5^
1.7620	208.0990	Cys-Ser	C_6_H_12_N_2_O_4_S	PP	7.35	0.00038
1.7771	147.0336	*O*-Acetyl-l-serine	C_5_H_9_NO_4_	AA	6.19	0.00068
**Group CD/Positive**						
0.9984	343.1224	Coenzyme B	C_11_H_22_NO_7_PS	-	2.54 × 10^−6^	3.37 × 10^−11^
1.0121	149.2115	Methionine	C_5_H_11_NO_2_S	AA/SM	0.57	0.00035
3.7640	227.1748	Pretyrosine	C_10_H_13_NO_5_	AA	89,506	1.12 × 10^−7^
0.7825	360.1494	Aldosterone	C_21_H_28_O_5_	LL	87,489	1.23 × 10^−8^
8.2828	229.1543	Pro-Asn	C_9_H_15_N_3_O_4_	PP	5852.70	2.76 × 10^−7^
8.7424	245.1130	β-Alanyl-l-arginine	C_9_H_19_N_5_O_3_	-	693.81	245.11300
0.8106	219.0260	*O*-Succinylhomoserine	C_8_H_13_NO_6_	AA	0.001788	219.02600
0.7955	203.0515	Lys-Gly	C_8_H_17_N_3_O_3_	PP	0.013665	1.92 × 10^−9^
1.0017	145.0471	2-Oxoglutaramate	C_5_H_7_NO_4_	AA	0.03	9.12 × 10^−9^
0.9794	85.0226	Piperidine	C_5_H_11_N	AA	0.34	1.46 × 10^−6^
**Group CD/Negative**						
0.8057	59.0112	Trimethylamine	C_3_H_9_N	EG	0.05	1.41 × 10^−7^
0.6974	79.9565	Pyrimidine	C_4_H_4_N_2_	NT/AA/CH	34,677	0.00034
2.4645	103.0543	*N,N*-Dimethylglycine	C_4_H_9_NO_2_	AA	0.000495	3.01 × 10^−5^
0.8080	119.0356	l-Homoserine	C_4_H_9_NO_3_	AA	0.03	6.21 × 10^−11^
3.0080	119.0507	Threonine	C_4_H_9_NO_3_	AA	45,026	0.00058
4.8198	121.0595	l-Cysteine	C_3_H_7_NO_2_S	AA	4.29	5.07 × 10^−6^
0.8044	125.0252	5-Methylcytosine	C_5_H_7_N_3_O	AA/SM	0.07	1.95 × 10^−6^
1.0160	129.0206	4-Oxoproline	C_5_H_7_NO_3_	AA	0.30	1.53 × 10^−6^
0.7957	131.0359	l-Leucine	C6H13NO2	AA	18.69	8.05 × 10^−5^
1.7654	147.0343	*O*-Acetyl-l-serine	C_5_H_9_NO_4_	AA	12.01	0.00037
0.7748	161.0462	*O*-Acetyl-l-homoserine	C_6_H_11_NO_4_	AA	0.35	1.09 × 10^−5^
1.7504	162.0563	Gly-Ser	C_5_H_10_N_2_O_4_	PP	31,159	7.46 × 10^−5^
2.9981	165.0566	l-Phenylalanine	C_9_H_11_NO_2_	AA/TL	1,200,100	0.00061
1.0140	177.0224	*N*-Formyl-l-methionine	C_6_H_11_NO_3_S	AA	13.71	7.75 × 10^−5^
1.5544	181.0513	Tyrosine	C_9_H_11_NO_3_	AA	0.44	0.00051
4.4615	193.0874	Phenylacetylglycine	C_10_H_11_NO_3_	AA	1062.60	0.00031
0.7555	202.9701	Cystathionine	C_7_H_14_N_2_O_4_S	AA	57,753	0.00044
1.6778	208.0980	Cys-Ser	C_6_H_12_N_2_O_4_S	PP	3.77	5.36 × 10^−5^
1.6254	222.0797	Gly-Phe	C_11_H_14_N_2_O_3_	PP	5.03	0.00029
5.0840	226.0190	Carnosine	C_9_H_14_N_4_O_3_	AA	14.70	226.01900
0.8045	236.0386	Ser-Met	C_8_H_16_N_2_O_4_S	PP	53,541	0.00051
2.9149	242.0137	Thymidine	C_10_H_14_N_2_O_5_	NT	0.12	8.74 × 10^−5^
1.7009	252.0893	Pro-His	C_11_H_16_N_4_O_3_	PP	111.38	7.03 × 10^−9^
2.5898	263.1405	Met-Asn	C_9_H_17_N_3_O_4_S	PP	568.34	0.00035
0.7992	269.0884	His-Asn	C_10_H_15_N_5_O_4_	PP	0.06	3.72 × 10^−5^
2.8149	283.0847	Gln-His	C_11_H_17_N_5_O_4_	PP	151,190	0.00049
1.6477	293.1519	Lys-Phe	C_15_H_23_N_3_O_3_	PP	73.11	0.00022
0.8082	341.1089	His-Trp	C_17_H_19_N_5_O_3_	PP	0.001072	3.93 × 10^−12^
**Group EF/Positive**						
0.7924	133.0260	Aspartic acid	C_4_H_7_NO_4_	-	109.94	0.00018
0.8467	163.0592	*N*-Acetyl-l-cysteine	C_5_H_9_NO_3_S	-	2.94	0.00824
3.9198	187.0960	*N*-Heptanoylglycine	C_9_H_17_NO_3_	-	6.41	1.18 × 10^−8^
0.9499	193.0341	Phenylacetylglycine	C_10_H_11_NO_3_	-	106.36	9.63 × 10^−5^
0.9514	210.0607	d-Glucaric acid	C_6_H_10_O_8_	-	608.64	0.00012
1.9229	232.1542	Asp-Val	C_9_H_16_N_2_O_5_	PP	4.48	2.74 × 10^−8^
0.9217	235.0874	Cys-Asn	C_7_H_13_N_3_O_4_S	PP	1.76 × 10^−5^	0.00808
3.9316	246.1701	Leu-Asp	C_10_H_18_N_2_O_5_	PP	6.99	4.74 × 10^−9^
0.7986	248.0219	Asp-Asp	C_8_H_12_N_2_O_7_	PP	214,930	0.00021
0.9859	254.1610	His-Val	C_11_H_18_N_4_O_3_	PP	0.08	0.00063
14.5615	274.2738	Lys-Lys	C_12_H_26_N_4_O_3_	PP	6.13 × 10^−7^	0.00424
0.8153	293.0983	Gln-Phe	C_14_H_19_N_3_O_4_	PP	75376	0.001205
0.8302	360.1497	Trp-Arg	C_17_H_24_N_6_O_3_	PP	11.74	3.78 × 10^−5^
**Group EF/Negative**						
0.7839	89.0235	l-Alanine	C_3_H_7_NO_2_	AA/SM/TL	1.06 × 10^−5^	0.00079
0.7404	95.9509	Methanesulfonic acid	CH_4_O_3_S	EG	1387.50	1.19 × 10^−5^
0.6579	107.9440	Benzoquinone	C_6_H_4_O_2_	SM	4959.20	4.51 × 10^−6^
0.9486	111.0112	Cytosine	C_4_H_5_N_3_O	NT	3.65	6.50 × 10^−5^
1.6075	115.0384	l-Proline	C_5_H_9_NO_2_	AA/SM	5.53	1.06 × 10^−5^
1.5504	117.0549	l-Valine	C_5_H_11_NO_2_	AA/TL/SM	20.24	3.07 × 10^−7^
1.6300	119.0499	l-Homoserine	C_4_H_9_NO_3_	AA	2.02 × 10^−5^	0.00039
0.9670	129.0186	4-Oxoproline	C_5_H_7_NO_3_	AA	6128	7.20 × 10^−8^
0.7902	146.9834	Glutamic acid	C_5_H_9_NO_4_	-	1.34 × 10^−5^	8.08 × 10^−5^
0.6532	152.9172	3-Sulfino-l-alanine	C_3_H_7_NO_4_S	-	106,100	0.00022
1.7560	160.0406	d-Alanyl-d-alanine	C_6_H_12_N_2_O3	-	4.43 × 10^−5^	0.00197
0.7889	161.0465	*O*-Acetyl-l-homoserine	C_6_H_11_NO_4_	-	0.21	0.00013
0.9159	211.0028	Phosphocreatine	C_4_H_10_N_3_O_5_P	-	51.19	0.00298
1.3969	218.1037	Thr-Val	C_9_H_18_N_2_O_4_	PP	0.26	0.00024
1.7541	239.9973	l-Cystine	C_6_H_12_N_2_O_4_S_2_	-	<0.01	0.00303
0.7222	291.0835	Ser-Trp	C_14_H_17_N_3_O_4_	PP	89.85	5.05 × 10^−5^
10.5696	293.1765	Lys-Phe	C_15_H_23_N_3_O_3_	PP	0.001526	0.00025
0.7730	426.9988	Adenosine diphosphate	C_10_H_15_N_5_O_10_P_2_	-	6227.70	7.00 × 10^−5^

Notes: RT, retention time; *m/z*, the measured *m/z*; MF, molecular formula; MP, Metabolic pathway; AA, Amino acid metabolites; PP, peptides; EG, energy metabolites; LL, lipid metabolites; NT, nucleotide metabolites; SM, biosynthesis of secondary metabolites; MT, membrane transport; CV, metabolism of cofactors and vitamins; CH, carbohydrate metabolism; TL, translation; “-”, unknown pathway.

## Supplementary Materials

The following are available online, Figure S1: BPI chromatograms for groups AB in ESI^+^/ESI^−^ scanning modes, Figure S2: BPI chromatograms for groups CD in ESI^+^/ESI^-^ scanning modes, Figure S3: BPI chromatograms for groups EF in ESI^+^/ESI^-^ scanning modes, Figure S4: Orthogonal partial least squares discriminant analysis (OPLS-DA) : S-plots of the data for three groups of samples (**AB**, **CD**, **EF**) obtained in ESI^-^ ion mode.
