# Supplementary material for: A Metabolomics Approach Uncovers Differences between Traditional and Commercial Dairy Products in Buryatia (Russian Federation)
Source: Molecules. 2018 Mar 22;23(4):735. doi: 10.3390/molecules23040735 (PMC6017790; doi:10.3390/molecules23040735)
Supplement: Supplementary file 1 [file molecules-23-00735-s001.pdf]

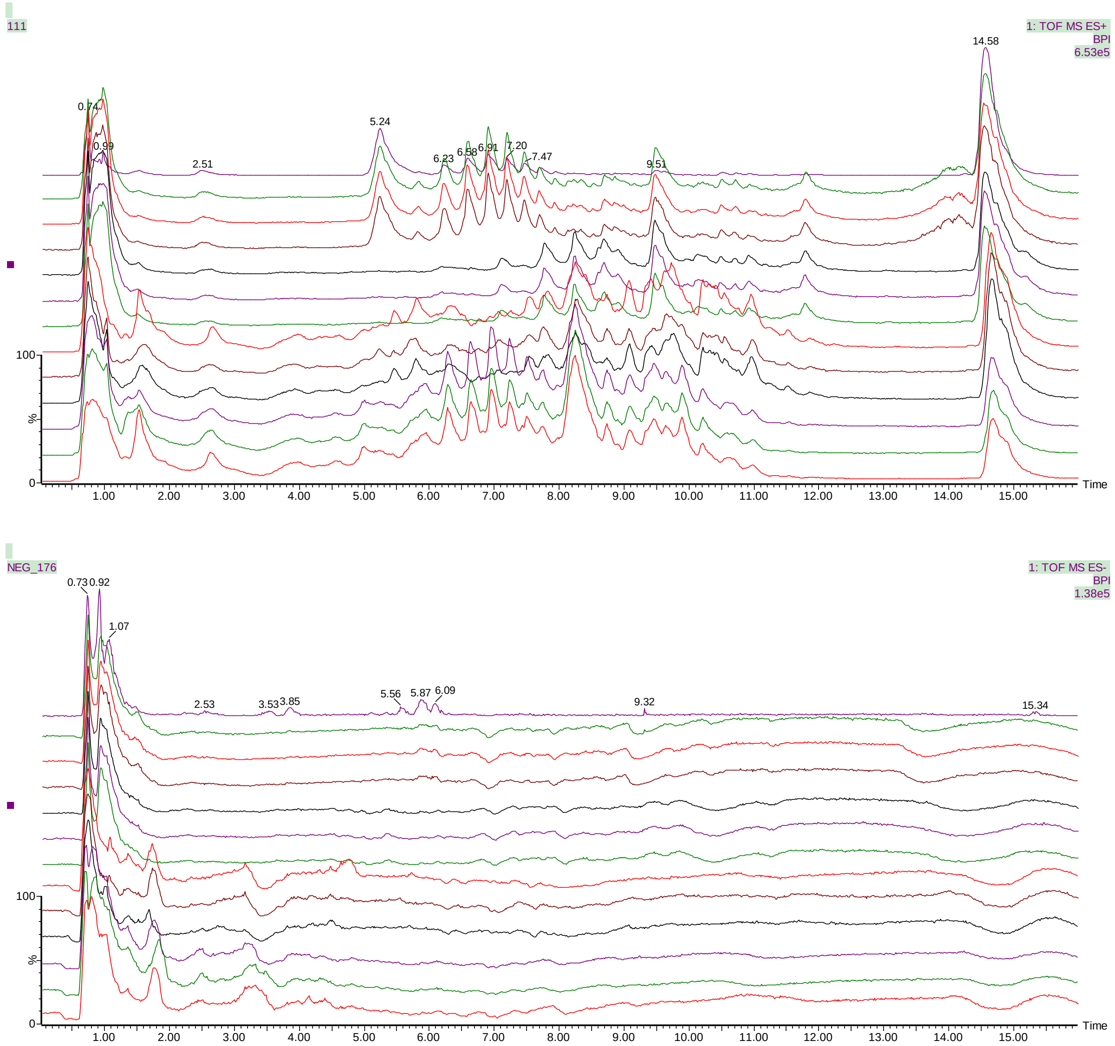

Supplemental Figure 1. BPI chromatograms for groups AB in ESI^{+}/ESI^{-} scanning modes

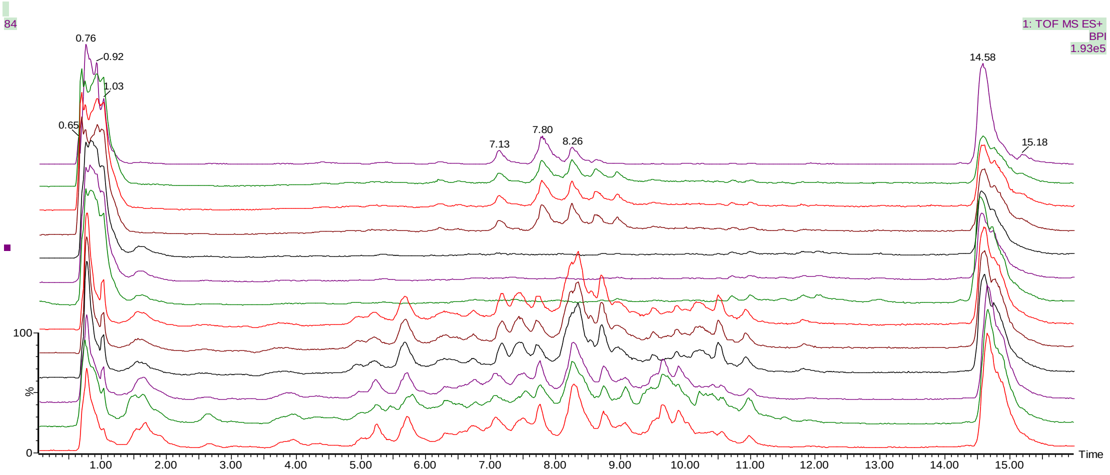

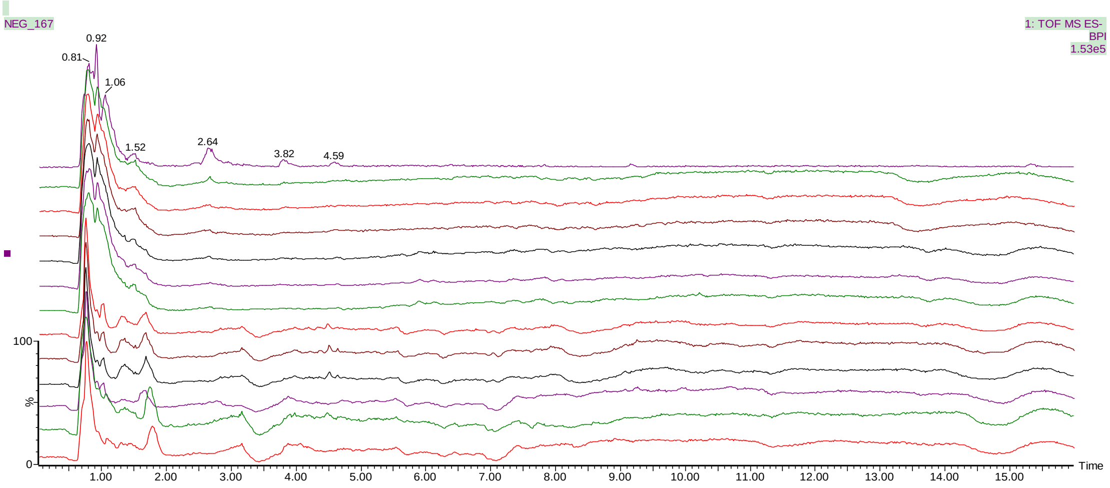

Supplemental Figure 2. BPI chromatograms for groups CD in ESI^+/ESI^- scanning modes

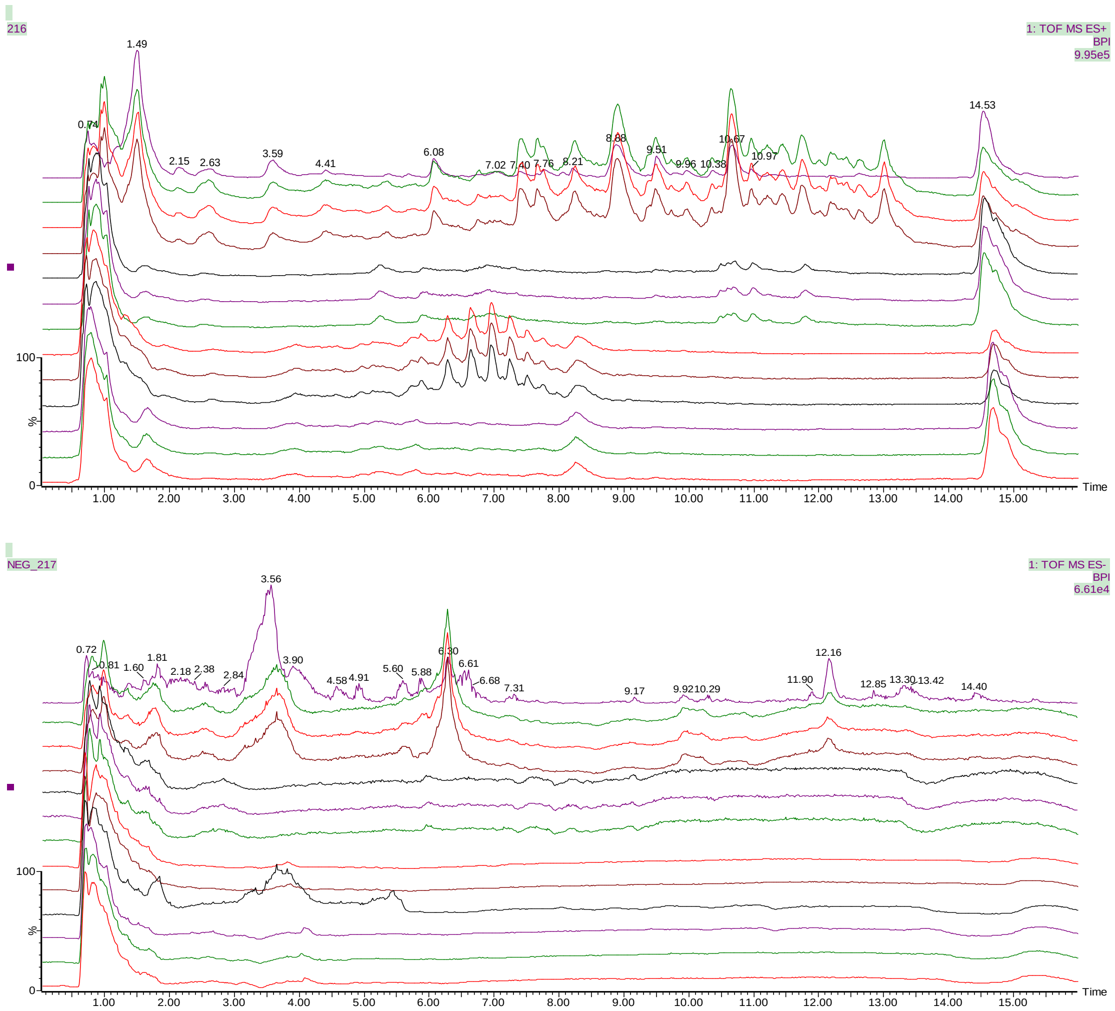

Supplemental Figure 3. BPI chromatograms for groups EF in ESI^{+}/ESI^{-} scanning modes

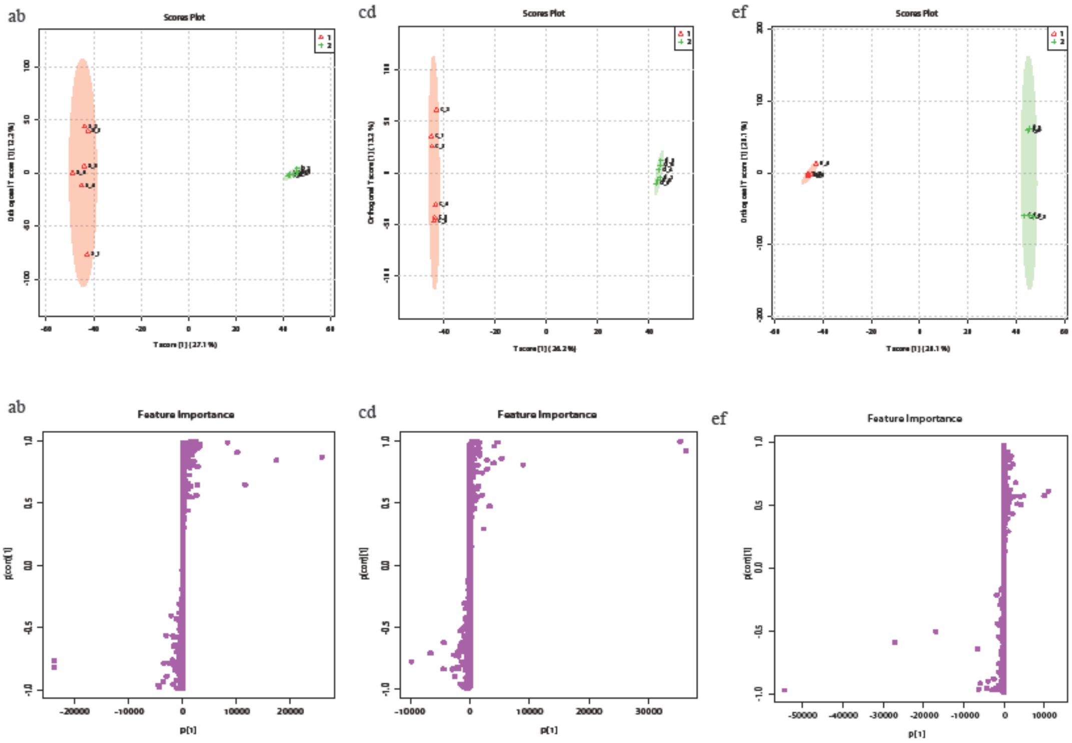

Figure S4: Orthogonal partial least squares discriminant analysis (OPLS-DA) and S-plots of the data for three groups of samples (AB, CD, EF) obtained in ESI$^-$ ion mode Figure S2:
